# An Optical Fiber Sensor for Axial Strain, Curvature, and Temperature Measurement Based on Single-Core Six-Hole Optical Fiber

**DOI:** 10.3390/s22041666

**Published:** 2022-02-21

**Authors:** Yujian Li, Changyuan Yu, Ping Lu

**Affiliations:** 1 The Department of Electronic and Information Engineering, The Hong Kong Polytechnic University, Hong Kong 999077, China; yujianwy.li@connect.polyu.hk; 2 National Engineering Laboratory for Next Generation Internet Access System, School of Optical and Electronic Information, Huazhong University of Science and Technology, Wuhan 430074, China; pluriver@mail.hust.edu.cn

**Keywords:** single-core six-hole optical fiber, strain, temperature, curvature, simultaneous measurement

## Abstract

In this paper, the field distribution and effective refractive index of transmission modes in single-core six-hole optical fiber were researched by modeling and simulation experiments. Based on the simulation results, a new type of sensor for axial strain, curvature, and temperature applications measurement was designed and fabricated. The experimental results showed that the axial strain sensitivities at different dips were −0.97 pm/με and −1.05 pm/με in the range from 0 to 2000 με, and the temperature sensitivities were 35.17 pm/°C and 47.27 pm/°C in the range from 25 to 75 °C. In addition, the proposed sensor also detected the curvature change with sensitivities of 7.36 dB/m^−^^1^ and 20.08 dB/m^−1^ from −2.582 m^−1^ to −1.826 m^−1^, respectively. Finally, through theoretical analysis, it can be deduced that this has potential application in the field of simultaneous measurement of strain and temperature.

## 1. Introduction

Compared to electronic sensors, optical sensors attracted the attention of researchers as soon as they appeared because of their advantages of high sensitivity [1], compact size [2], anti-electromagnetic interference [3], and low cost [4]. Among the optical sensors, the optical fiber sensors are the most commonly used in various application scenarios because optical fiber is not only the sensing element but also the main transmission unit in the whole communication system [5]. Thus, the optical fiber sensors play an important role in many physical parameter monitoring systems, such as axial strain and curvature measurement in large buildings, marine structure health monitoring systems, and temperature and curvature measurements in vital sign monitoring systems. Physical parameter sensors were fabricated on single-mode fibers (SMFs) using a wide range of different types of schemes during previous years. For instance, a taper structure fabricated by an oxyhydrogen flame has been used for measuring axial strain and temperature; the miniature ultra-long period fiber prating (LPFG) and fiber Bragg grating (FBG) were designed for simultaneous measurement of axial strain and temperature [6,7]; and inline Fabry-Perot (FP) was designed for simultaneous measurement of high temperature and strain [8]. Two micro-notches were inscribed in SMF through effective CO_2_ laser illumination for fabricating a bending vector sensor [9]; the sensitivity to curvature change of a lateral offset splicing structure was demonstrated by Zhang et al. [10].

However, the mechanical strength of the taper structure and micro-notch is very low because it always is produced by a high-temperature process. Similarly, the inline FP also faces the same problem because of the air cavity in the fiber [11,12,13], and the cost of the grating fabrication is very high. With the development of optical fiber preparation technology, various micro-structural fibers(M-SF) have been developed for fabricating new types of sensors to make up for the shortcomings of the sensors based on SMF. For example, a highly sensitive fiber loop ringdown strain sensor was fabricated using a photonic crystal fiber (PCF) interferometer [14], the Mach-Zehnder interferometer in multicore fiber (MCF) was combined with a helical structure for highly sensitive strain measurement [15], a refractive index and strain sensor were proposed based on twin-core fiber (TCF) with a novel T-shaped taper [16], and hollow-core fiber (HCF) was tapered for strain sensing [17]. Polymer optical fiber (POF) was designed and applied by a torque sensor in 2018 [18]. Similarly, the mechanical strength of the micro-structure-based sensor is also very low because of the collapsed region between the SMF and the M-SF [19]. In addition, most of the M-SF-based sensors can only detect no more than two physical parameters. Therefore, multi-parameter sensors with low cost, robust mechanical structures, and wide measurement ranges have considerable research value.

Based on the demand, we designed an optical fiber sensor by using a new type of special microstructure optical fiber and proved its feasibility by experiments. During the process of the sensor design, we researched the mode field distribution of the single-core six-hole microstructure optical fiber (SCSHF) by simulation calculations. It can be induced from the results that an inline Mach-Zehnder interference (MZI) is formed if the fundamental core mode (FCM) and the high-order cladding modes (H-OCM) are excited at the same time. Then, the fabrication parameters of the sensor were designed by simulation and actual experiments. The simple preparation process and low preparation cost make the sensor suitable for quantity production. The sensing performance of the SCSHF sensor was also researched. The strain sensitivities of the sensor at different dips were 0.97 pm/με and 1.05 pm/με from 0 to 2000 με, respectively. The temperature sensitivities were 35.17 pm/°C and 47.27 pm/°C from 25 to 75 °C, respectively. The curvature sensitivities were 7.36 dB/m^−1^ and 20.08 dB/m^−1^ in the range from −2.582 m^−1^ to −1.826 m^−1^, respectively. Through theoretical analysis, it can be found that temperature and axial strain changes can be monitored at the same time by calculating the offset of the sensor spectrum, which means the temperature cross-sensitivity can be compensated.

## 2. Fabrication and Principles

### 2.1. Fabrication

**Figure 1:**
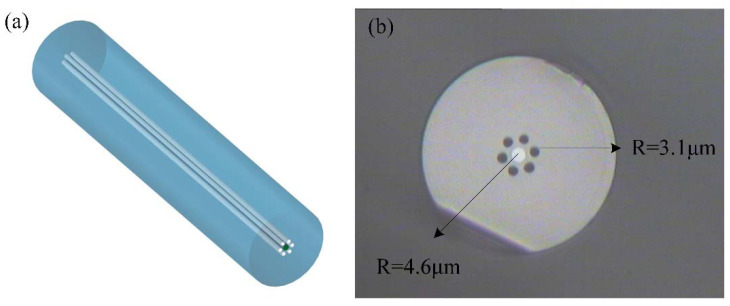
(**a**) Structural diagram of SCSHF; (**b**) cross-section of SCSHF under a microscope.

The SCSHF fiber was designed and fabricated by Yangtze Optical Electronic Co., Ltd. (YOEC), which is an optical fiber design and manufacturing company in Wuhan, China. The structure and cross-section of the SCSHF are shown in Figure 1. Different from the SMF, there are six air holes evenly distributed around the core in the cladding of the fiber, which may help promote the sensing sensitivity. The radius of the core is ~4.6 μm and the radius of the six air holes is ~3.1 μm. The air holes do not cause severe collapse during the fusing process since the radius of the air holes is less than the radius of the cladding. Thus, the air holes do not influence the mechanical strength of the sensor.

**Figure 2:**
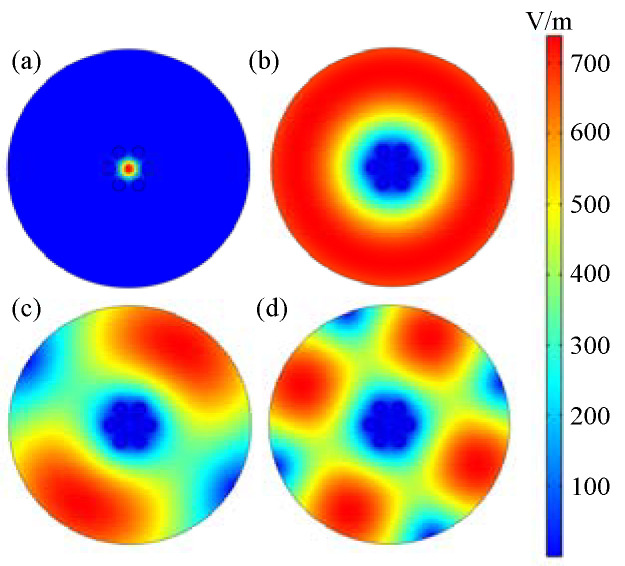
Simulation results of transmission mode in SCSHF; (**a**) FCM; (**b**–**d**) H-OCM.

To understand the mode field distribution in optical fiber, simulation calculations were conducted and the results are shown in Figure 2. Figure 2a is the FCM transmitted in the SCSHF optical fiber and its effective refractive index (EFI) is 1.4476. The mode field distribution of H-OCM is shown from Figure 2b–d. Except that the fundamental mode can propagate through the core, other high-order modes can only be transmitted in the cladding region. Thus, if the FCM and H-OCM are excited at the same time and then coupled together, an inline MZI is produced because of the phase difference between them. If the external environment parameters fluctuate, the phase difference also changes, resulting in the change in spectrum. Thus, the SCSHF may have a potential application for sensing.

**Figure 3:**
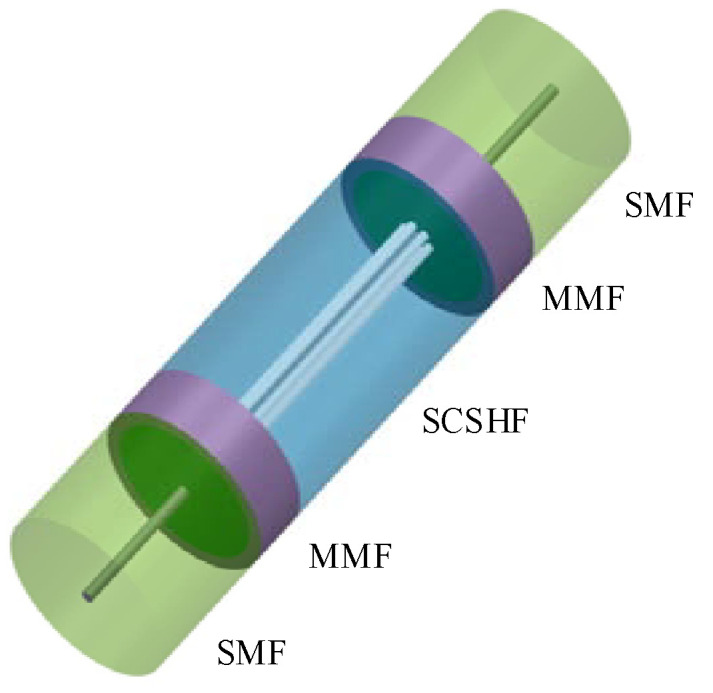
Structural diagram of the sensor based on SCSHF.

**Figure 4:**
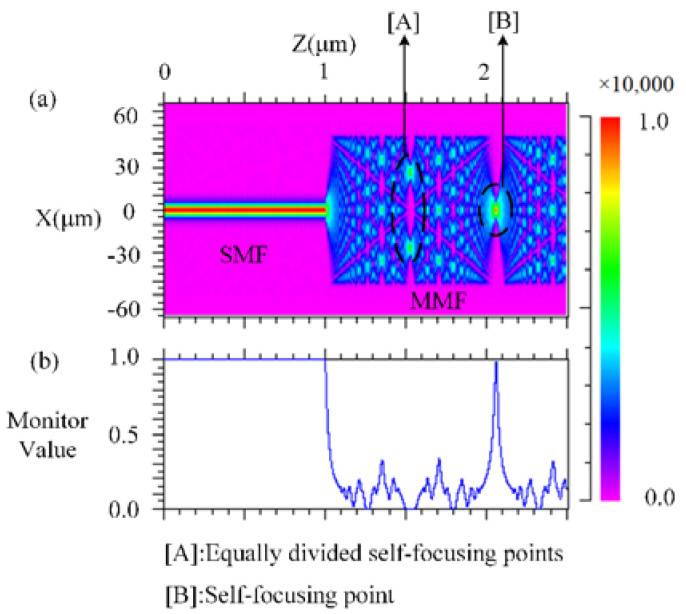
(**a**) Optical field distribution in the SM structure; (**b**) relative value of light intensity in the core of SMF and MMF.

According to the inline MZI theory, the sensor shown in Figure 3 was designed. Two sections of multi-mode fiber (MMF) were fused between SMF and SCSHF. The MMF was employed to enlarge the mode field for exciting the modes in the core and cladding to make sure that enough modes participated in the interference. To find the proper length of the MMF, a simulation experiment based on SM structure was conducted and the results are shown in Figure 4a,b. The dimension of the modeling is 2D because of the circular symmetry of the SM structure. The simulation parameters are shown in the Table 1.

The self-focusing effect occurs when the light travels from the SMF to the MMF. The light is equally divided into two parts at point A, while the light is focused together at point B. The blue line in Figure 4b is the relative value of the light intensity at the center point of the fiber. The value is 0 at point A, while the value is 1 at point B. The length of point A is about 550 μm and the length of point B is 1100 μm. Both points cannot simultaneously excite modes in the core and cladding. Thus, the proper distance of the MMF may be from 350 μm to 500 μm. Taking into account the accuracy limitations of the fiber cleaver, the final length of the MMF was designed as 400 μm.

**Figure 5:**
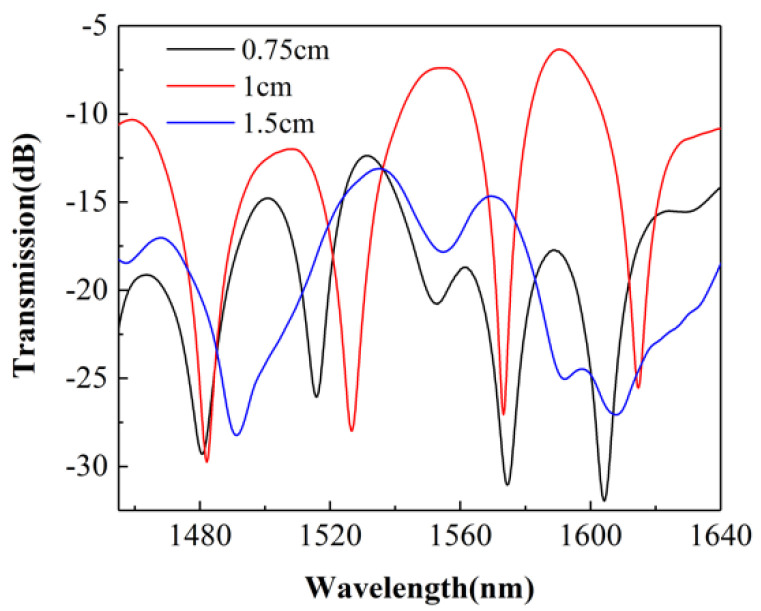
Transmitted spectra of the sensor with different lengths of SCSHF.

The length of the SCSHF was determined by various experiments and the result is shown in Figure 5. If the length of the SCSHF is too long, the excited cladding modes will leak out, resulting in the contrast of interference fringes decreasing as the blue line in Figure 5 shows. On the contrary, if the length of SCSHF is too short, there will be many cladding modes participating in the MZI, which may result in a high-complexity spectrum as the black line in Figure 5 shows. Then, the difficulty of the demodulation process will rise accordingly. From Figure 5, it is obvious that the final spectrum of the sensor is close to the spectrum of two-beam lights interference when 1 cm SCSHF is fused between the MMFs.

**Figure 6:**
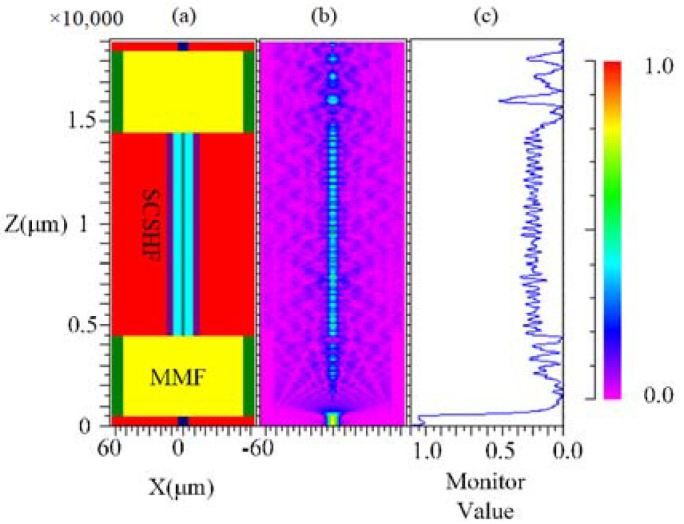
(**a**) Simulation model of the SCSHF sensor; (**b**) Optical field distribution in the SCSHF sensor; (**c**) Relative value of light intensity in the SCSHF sensor.

Figure 6a is the simulation model of the SCSHF sensor built in the simulation soft and the simulation tool is Beam PROP. The dimension of the modeling is 3D because the structure of the SCSHF cannot be simplified. The simulation parameters are shown in the Table 2.

Figure 6b shows the energy distribution of the light field in the SCSHF sensor. The light power along the *Z*-axis shows an obvious alternating distribution of strength and weakness. The peak of the blue line in Figure 6c corresponds to the maximum interference light intensity, while the dip corresponds to the minimum interference light intensity. The simulation results indicate that the inline MZI is produced in the SCSH sensor, which is consistent with theoretical analysis.

### 2.2. Principles

**Figure 7:**
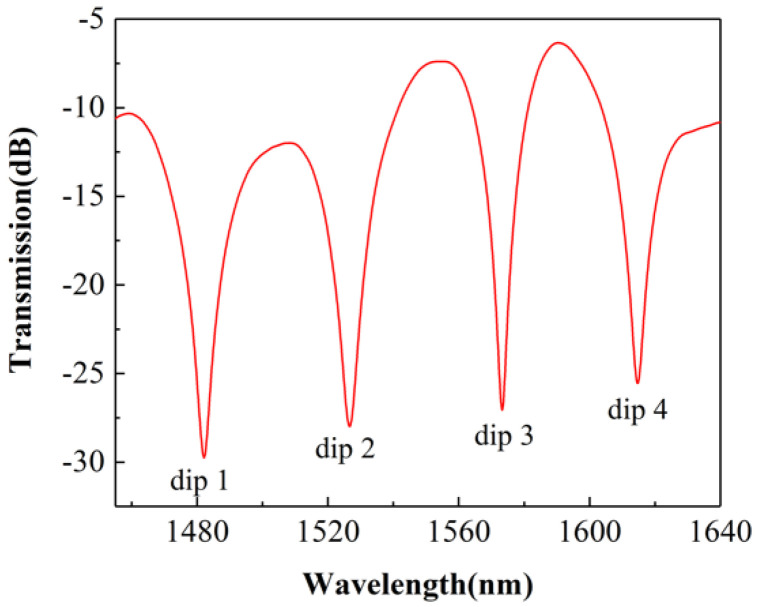
Spectrum of the SCSHF sensor.

The red line in Figure 7 is the spectrum of the SCSHF sensor obtained from the experiment. Four dips can be observed in the spectrum at wavelengths 1482.1, 1526.7, 1573.3, and 1614.7 nm, respectively. All of the four dips can be used for physical parameter sensing.

When the input light is transmitted through the sensor, the light is divided into three parts: the light transmitted in the fiber core, the light transmitted in the fiber cladding, and the light leaked out of the sensor.

(1) {α2=IcoIβ2=IclIγ2=IraI

I_co_ is the intensity of the FCM, I_cl_ is the intensity of cladding mode, and I_ra_ is the intensity of the leaking mode. α, β, and γ are the proportionality coefficients and the relationship between them is α^2^ + β^2^ + γ^2^ = 1.

For simplifying the interference modal, only the interference between the FCM and one cladding mode is considered. Thus, the transmitted light intensity of the SCSHF sensor I_out_ can be calculated by

(2) $$I_{\mathrm{out}}{=I}_{\mathrm{co}}{+I}_{\mathrm{cl}}+2\sqrt{I_{\mathrm{co}}I_{\mathrm{cl}}}\varphi$$

where I_out_ is the intensity of the output light, and φ represents the phase difference between the core and cladding mode, which is related to the effective refractive index of the mode and the distance of mode coupling. The expression for φ is as follows:

(3) $$\varphi =2\pi \frac{n_{\mathrm{eff}}^{\mathrm{co}}L_{\mathrm{co}}-n_{\mathrm{eff}}^{\mathrm{cl}}L_{\mathrm{cl}}}{\lambda }$$

Here, $n_{eff}^{co}$ and $n_{eff}^{cl}$ are the EFI of the FCM and the cladding mode, respectively. L_co_ and L_cl_ are the distances of mode transmission.

I_out_ achieves the maximum value I_max_ when φ equals 2mπ. I_out_ achieves the minimum value I_min_ when φ equals 2(m + 1)π, indicating a dip in the spectrum. λ_dip_ can be calculated as:

(4) $$\lambda_{\mathrm{dip}}=\frac{2}{2m+1}{(n}_{\mathrm{eff}}^{\mathrm{co}}L_{\mathrm{co}}-n_{\mathrm{eff}}^{\mathrm{cl}}L_{\mathrm{cl}})$$

The contrast of the interference fringe K can be expressed as:

(5) $$K=\frac{I_{\max}-I_{\min}}{I_{\max}+I_{\min}}$$

Combining the Equations (1), (2), and (5), K can be calculated as:

(6) K=2αβα2+β2=2(αβ)1+(αβ)2

On account of the elastic optical effect (EOE) [20], the EFI of the transmitted mode changes with the fluctuation of the strain exerting on the sensor, resulting in the dip wavelength shifting. The strain sensitivity induced by the length variation of the sensor is several orders of magnitude smaller than that induced by the elastic optical effect, so it can be ignored. Then, the strain-wavelength sensitivity can be expressed as:

(7) $$\frac{{d\lambda }_{\mathrm{dip}}}{\mathrm{dv}}=\frac{2}{2m+1}(\frac{{\mathrm{dn}}_{\mathrm{eff}}^{\mathrm{co}}}{\mathrm{dv}}L_{\mathrm{co}}-\frac{{\mathrm{dn}}_{\mathrm{eff}}^{\mathrm{cl}}}{\mathrm{dv}}L_{\mathrm{cl}})$$

As for the temperature measurement, the wavelength shifting may result from the thermos-optic effect and the thermal expansion effect [21]. The temperature wavelength sensitivity can be expressed as:

(8) $$\frac{{d\lambda }_{\mathrm{dip}}}{\mathrm{dT}}=\frac{2}{2m+1}[\left(\frac{{\mathrm{dn}}_{\mathrm{eff}}^{\mathrm{co}}}{\mathrm{dT}}L_{\mathrm{co}}-\frac{{\mathrm{dn}}_{\mathrm{eff}}^{\mathrm{cl}}}{\mathrm{dT}}L_{\mathrm{cl}}\right)+\left(n_{\mathrm{eff}}^{\mathrm{co}}\frac{{\mathrm{dL}}_{\mathrm{co}}}{\mathrm{dT}}-n_{\mathrm{eff}}^{\mathrm{cl}}\frac{{\mathrm{dL}}_{\mathrm{cl}}}{\mathrm{dT}}\right)]$$

When the sensor is bent, a part of the cladding light leaks into the air and becomes leaking light, while the core light has no change. That is, α has no change but β becomes smaller. According to Equation (6), the contrast of the interference fringe K also becomes smaller.

## 3. Experimental Results

### 3.1. Strain Sensing Experiment

**Figure 8:**
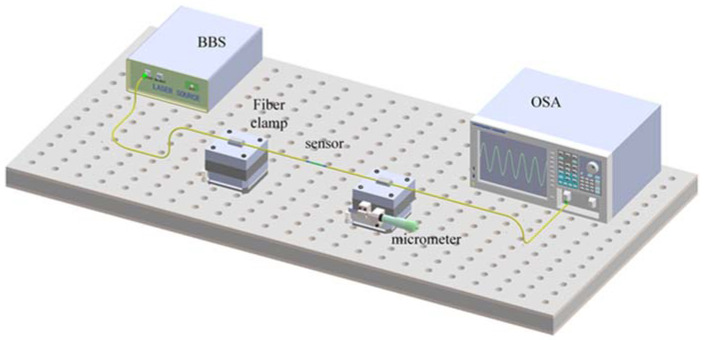
Measurement system for strain sensing.

Figure 8 shows the experimental devices used in the strain sensing experiment. The input light is imported by a broadband light source (BBS). The output spectrum is recorded by an optical spectrum analyzer (YOKOGAWA, Tokyo, Japan, AQ6070D). The sensor is fixed on the 3-D moving platform and the distance L_0_ between the two platforms is 20 cm. The distance can be adjusted by the micrometer with a fixed step of 0.01 mm. Thus, the axial strain applied on the sensor can be changed by making L_0_ grow. The micro-strain change from every move step can be calculated by ∆v = ∆L/L_0_ = 50 με.

**Figure 9:**
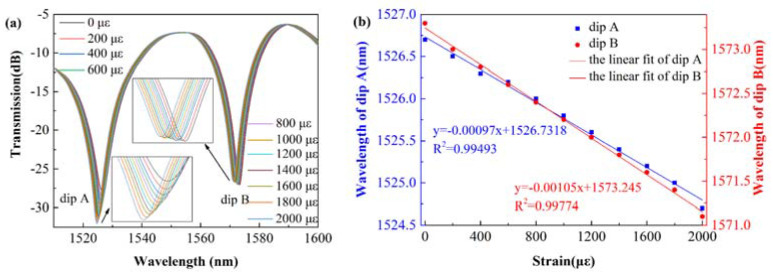
(**a**) Transmitted spectra of the SCSHF sensor under different axial strains; (**b**) linear fitting curve between dip wavelength and axial strain.

The different color lines in Figure 9a are the spectra from 0 με to 2000 με with the step of 200 με. From the illustration in Figure 9a, the transmitted spectrum shows an obvious drift to short wavelength with the strain increasing. Figure 9b shows the linear fit curve between the dip wavelength and the axial strain. The R-Squared values of the linear fit at dip A and dip B are 0.99493 and 0.99774, respectively, meaning that the dip wavelength offset is a linear function of the strain change. The strain-wavelength sensitivity of dip A is −1.05 pm/με and the strain-wavelength sensitivity of dip B is −0.97 pm/με, which are obtained from the line slope.

### 3.2. Curvature Sensing Experiment

**Figure 10:**
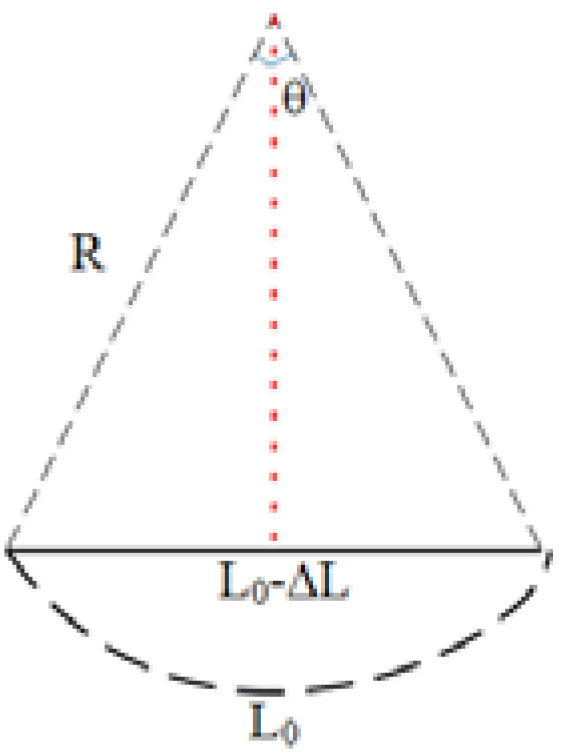
Schematic diagram of curvature change of the sensor.

The curvature sensing experiment is also conducted by the system in Figure 8. By shortening the distance between the two clamps, the curvature added to the sensor is changed. According to Figure 10, the curvature can be calculated by the following equation.

(9) $$\mathrm{Rsin}\left(\frac{L_{0}}{2R}\right)=\frac{L_{0}-\Delta L}{2}$$

**Figure 11:**
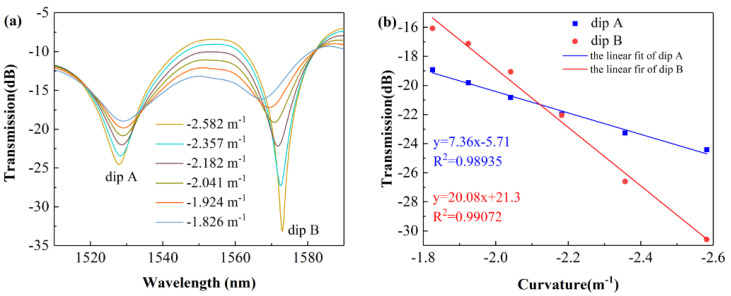
(**a**) Transmitted spectra of the SCSHF sensor under different curvatures; (**b**) linear fitting curve between dip intensity and curvature.

From Figure 11a, it can be seen that the contrast of the interference fringe becomes smaller as the two fiber clamps get closer, which is consistent with the theoretical analysis in Section 2.2. Figure 11b shows the linear fitting curves between dip intensity and curvature at dip A and B; the R-Squared values of the linear fit at dip A and dip B are 0.98935 and 0.99072, respectively. From the slope of the fitting curve, it can be seen that the sensor responds linearly to the curvature change with sensitivities of 7.36 dB/m^−1^ and 20.08 dB/m^−1^ from −2.582 m^−1^ to −1.826 m^−1^, respectively.

### 3.3. Temperature Sensing Experiment

**Figure 12:**
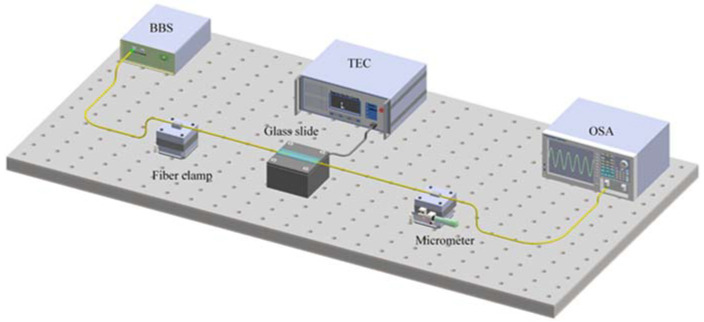
Measurement system for temperature sensing.

As shown in Figure 12, light source was also BBS and the spectrum monitoring equipment was also OSA. A high-precision digitally controlled Thermo-Electric Cooler (TEC, TLTP-TEC0510, Wuhan Talent Century Technology Co., Ltd, Wuhan, China) was used as the heating source. To eliminate the cross-sensitivity of the surrounding environment, the sensor was fixed on the 3D moving platform by two fiber claps to keep it at a horizontal level. The role of the slide was to ensure a uniform temperature distribution around the sensor.

**Figure 13:**
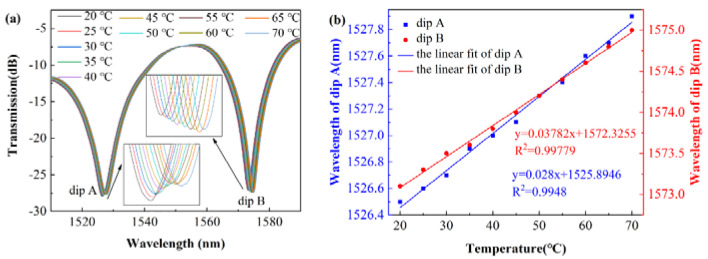
(**a**) Transmitted spectra of the SCSHF sensor under different temperatures; (**b**) linear fiting curve between dip wavelength and temperature.

For the temperature sensing experiment, the measurement range was from 25 °C to 75 °C by the fixed step of 5 °C and the optical spectra under different temperatures are shown in Figure 13a. Each spectrum was recorded 15 min after adjusting the temperature of TEC to stabilize the temperature. It is easy to find that the optical spectrum is red-shifted with increasing temperature. To further understand the SCSHF fiber sensor’s response characteristics to temperature, a linear fitting is conducted on the dip wavelength of A and B and the result is in Figure 13b. The sensitivity at different dips can be obtained from the slope of the fitting line. The R-Squared values of the linear fitting line at dip A and B are 0.99779 and 0.9948, respectively. Therefore, it can be concluded that the sensor responds linearly to temperature with a sensitivity of 37.82 pm/°C and 28 pm/°C from 25 to 75 °C.

### 3.4. Theoretical Analysis of Simultaneous Measurement

From the above results and discussion, it can be concluded that the dip wavelength of the SCSHF responds linearly to external temperature and microstrain while the intensity of the interference dip responds linearly to the curvature. Thus, the dip wavelength will also shift linearly if the temperature and strain in the environment around the sensor change at the same time. The amount of wavelength shift can be calculated by the following equation [22]:

(10) $$\Delta \lambda_{A}{=S}_{A}^{T}*\Delta {T+S}_{A}^{v}*\Delta v\;\Delta \lambda_{B}{=S}_{B}^{T}*\Delta {T+S}_{B}^{v}*\Delta v$$

where ∆v is the amount of strain change, ∆T is the amount of temperature change, $S_{A}^{v}$ and $S_{B}^{v}$ are the strain-wavelength sensitivities of dip A and B, and $S_{A}^{T}$ and $S_{B}^{T}$ are the temperature-wavelength sensitivities of dip A and dip B.

Equation (10) can be transformed into a matrix form as:

(11) $$\left[\begin{array}{c} \Delta \lambda_{A}\\ \Delta \lambda_{B} \end{array}\right]=\left[\begin{array}{cc} S_{A}^{T} & S_{A}^{v}\\ S_{B}^{T} & S_{B}^{v} \end{array}\right]\left[\begin{array}{c} \Delta T\\ \Delta v \end{array}\right]$$

Since the sensitivity of different cladding modes to the surrounding environment is quite different, there will be a sensitivity deviation between different dips. Then, the amount of temperature and strain changes can be obtained by matrix transformation.

(12) $$\left[\begin{array}{c} \Delta T\\ \Delta v \end{array}\right]={\left[\begin{array}{cc} S_{A}^{T} & S_{A}^{v}\\ S_{B}^{T} & S_{B}^{v} \end{array}\right]}^{-1}\left[\begin{array}{c} \Delta \lambda_{A}\\ \Delta \lambda_{B} \end{array}\right]$$

As for simultaneous measurement of curvature and temperature, the proposed sensor cannot finish this work because there will be cross-talk between the dip wavelength and intensity. However, the proposed sensor can be used in an acoustics sensing system, which can ignore the temperature change. For example, the sensor can be attached to the diameter position of a PET film. Then, the acoustic field information can be modulated to the curvature change of the SCSHF sensor by the vibration of the PET film. Finally, the acoustic field information can be demodulated by the system proposed in [23].

## 4. Discussion

Table 3 provides a comparison between SCSHF sensors with other optical fiber sensors on sensing performance. The main points of comparison are strain sensitivity, temperature sensitivity, curvature sensitivity, and temperature cross-sensitivity. From the table, it can be summarized that the proposed SCSHF sensor can detect three physical parameters with high sensitivities while the other sensors can only detect at most two parameters. Besides, the designed sensor can eliminate the temperature cross-sensitivity when it is used for simultaneous measurement of axial strain and temperature. The raw material for sensor fabrication is not expensive and the fabrication process is much easier than other sensors, which is in favor of mass production. The size of the proposed sensor is only 20 mm, which is useful for application in a miniaturization system. All the advantages above make the sensor have considerable potential for use in the health monitoring system of large architecture and many other fields.

## 5. Conclusions

In summary, we designed a new type of optical fiber sensor based on SCSHF. The fabrication parameters were obtained from the simulation experiments. Then, test systems were built to study its sensing responses to axial strain, curvature, and temperature. The sensor responds linearly to strain with a sensitivity of −0.97 pm/με and −1.05 pm/με from 0 to 2000 με. The temperature sensitivities of 37.82 pm/°C and 28 pm/°C from 25 to 75 °C and the curvature sensitivities of 7.36 dB/m^−1^ and 20.08 dB/m^−1^ from −2.582 m^−1^ to −1.826 m^−1^, respectively, were also obtained. Additionally, the sensor has the ability to eliminate temperature cross-sensitivity through mathematical calculations when the axial strain and temperature change at the same time. Compared to other types of strain sensors, the SCSHF sensor has some good points such as low cost, small size, and high sensitivity, giving it broad application prospects in engineering, medical, and many other fields.

## Figures and Tables

**Table 1:** Simulation parameters of SM structure.

	SMF	MMF
Core diameter	9.2 μm	105 μm
Cladding diameter	125 μm	125 μm
EFI of core	1.4509	1.4509
EFI of cladding	1.444	1.444
Wavelength	1.55 μm	1.55 μm

**Table 2:** Simulation parameters of SCSHF sensor.

	SMF	MMF	SCSHF
Core diameter	9.2 μm	105 μm	9.2 μm
Cladding diameter	125 μm	125 μm	125 μm
EFI of core	1.4509	1.4509	1.4509
EFI of cladding	1.444	1.444	1.444
Distance between air hole and core	-	-	10 μm
Length	50 μm	400 μm	1 cm
Wavelength	1.55 μm	1.55 μm	1.55 μm

**Table 3:** Comparison with other optical fiber sensors.

Structure	Strain Sensitivity (pm/με)	Temperature Sensitivity (pm/°C)	Curvature Sensitivity (dB/m^−1^)	Temperature Cross Sensitivity	Reference
FBG-MMF	0.12	9		low	[24]
TCF-TFBG	0.67	33		low	[25]
TC-PCF	0.31	6.68		low	[26]
HCPCF	−0.81	-		high	[27]
FMF-FBG	0.91	11.5		low	[28]
7CF-FBG		12	−7.28		[29]
SMF-PCF			8.35	low	[30]
Taper-FBG		10	0.1196		[31]
SCSHF	−1.05	37.82	20.08	low	This work

## Data Availability

The data presented in this study are available on request from the corresponding author. The data are not publicly available due to.
